# Interlayer‐Sliding‐Enabled Multiferroicity and Giant Switchable Anomalous Hall Conductivity in RuO_2_Zn_2_F_2_ Bilayer

**DOI:** 10.1002/advs.74923

**Published:** 2026-05-07

**Authors:** Djamel Bezzerga, Imran Khan, Ganie Suhail Ahmad, Jisang Hong

**Affiliations:** ^1^ Department of Physics Pukyong National University Busan South Korea

**Keywords:** anomalous hall conductivity, piezoelectricity, RuO_2_Zn_2_F_2_ bilayer, sliding ferroelectricity

## Abstract

Recently, a septuple‐atomic‐layer RuO_2_Zn_2_F_2_ semiconducting monolayer, which belongs to the novel 2D materials MA_2_Z_4_ family, has emerged as a suitable above‐room‐temperature ferromagnet. Based upon these findings, first‐principles calculations are performed to study the RuO_2_Zn_2_F_2_ bilayer. A competitive interplay between ferromagnetic (FM) and A‐type antiferromagnetic (AFM) ground states is found, indicating strong tunability of the magnetic phase. Moreover, the electronic band structures are sensitive to their magnetic order and stacking configuration. Particularly, the aligned‐stacking configuration induces an out‐of‐plane electric polarization of 1.35 pC/m, which can be efficiently switched via interlayer sliding with an ultra‐low energy barrier of 19.3 meV/unit cell, realizing sliding ferroelectricity. Furthermore, the sign of valley polarization reverses with the switching of the electric polarization in the AFM configuration. Besides, a pronounced switchable anomalous Hall conductivity exceeding ±250 S/cm is obtained in the AFM state, while a giant anomalous Hall conductivity of −527 S/cm is achieved in the FM state. Meanwhile, RuO_2_Zn_2_F_2_ bilayer exhibits colossal in‐plane (d_11_ = 8.13 pm/V) and significant out‐of‐plane (d_31_ = 0.61 pm/V) piezoelectric response. Consequently, the results establish RuO_2_Zn_2_F_2_ bilayer as a promising platform for slidetronic, valleytronic, and spintronic applications.

## Graphical abstract

Interlayer sliding in the RuO_2_Zn_2_F_2_ bilayer induces ferroelectricity and enables reversible valley polarization switching. The electric dipole and valley‐resolved band edges are intimately coupled, revealing sliding ferroelectricity as a powerful mechanism for electrical control of valley degrees of freedom in 2D materials.

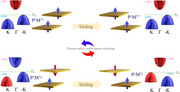

## 1 Introduction

Research on 2D materials has greatly expanded our understanding of quantum phenomena at the atomic level, opening new prospects for multifunctional device design [1, 2]. In particular, van der Waals (vdW) layered materials offer remarkable opportunities to control electronic, spin, and valley degrees of freedom, ultimately driving progress in spintronic, valleytronic, twistronic, and slidetronic applications [3, 4, 5, 6, 7]. Recently, a novel mechanism of ferroelectricity in 2D vdW materials, namely “sliding ferroelectricity”, was theoretically proposed [8] and later experimentally confirmed [9, 10, 11]. In 2D vdW materials such as stacked *h*‐BN bilayers or graphene/*h*‐BN heterostructures, the breakdown of centrosymmetry by relative layer stacking results in out‐of‐plane electric polarization, originally driven by interlayer charge redistribution and transfer [12, 13]. The relative arrangement of the layers dictates the amplitude and direction of electric polarization and can be altered by relative layer displacement, leading to “unconventional ferroelectricity” [14, 15]. This strategy has opened a new area in materials research known as slidetronics [16]. In the magnetic systems, magnetic ordering arrangements such as ferromagnetic (FM), antiferromagnetic (AFM), or altermagnetic (AM) are crucial for spintronic applications; spin filtering and magnetoelectric coupling [17, 18, 19]. Meanwhile, recent advances have enabled the successful synthesis of MA_2_Z_4_ (M: transition metal, A: group IV element, Z: group V element) [20]. This family is further expected to encompass transition‐metal oxides and halide‐functionalized systems, offering a broad platform for magnetic and electronic engineering in 2D heterostructures [21, 22]. For instance, it has been claimed that a 2D VSi_2_N_4_ bilayer has sliding ferroelectricity with A‐type antiferromagnetic ordering. [23]. Within this framework, the surface‐decorated RuO_2_ monolayers can be viewed as MA_2_Z_4_‐type derivatives, where functionalization stabilizes the ideal hexagonal RuO_2_ monolayer and tunes its electronic and magnetic behavior [20, 24]. Notably, the surface modifications of Ruthenium (Ru) atoms give rise to several stable RuO_2_‐based derivatives, including Ru(OLi)_2_ [25], RuO_2_Si_2_N_2_ [26], RuO_2_(MgF)_2_, and RuO_2_(ZnF)_2_ monolayers [27]. In particular, the RuO_2_(ZnF)_2_ monolayer is predicted to be an Ising‐type ferromagnet with ultrahigh Curie temperatures of 972 K. Due to the strong spin–orbit coupling (SOC), the hexagonal RuO_2_(ZnF)_2_ monolayer exhibits pronounced valley polarizations of up to 194 meV at the valence band maximum, as further confirmed by a four‐band k·p model. It is noteworthy that RuO_2_(ZnF)_2_ monolayer maintains an above‐room‐temperature Ising ferromagnetism under both hole doping and biaxial strains. The anomalous valley Hall effect can be naturally achieved in a system through doping moderate holes and infrared light irradiation upon applying an in‐plane electric field. Also, the spin and valley polarizations can be inverted via compressive strain and magnetization flipping. These outstanding attributes expose the vast potential of RuO_2_(ZnF)_2_ monolayer for a broad range of potential applications in straintronics, spintronics, valleytronics, optoelectronics, and their integration. Although the RuO_2_(ZnF)_2_ monolayer lacks intrinsic ferroelectricity due to symmetry constraints, we investigate sliding ferroelectricity in its bilayer form and the potential emergence of multiferroicity. Note that the ferroelectricity in ferromagnetic TcIrGe_2_S_6_ bilayers originates from Ge ionic shifting [28] and ion‐cation displacement in CrInSe_3_/In_2_S_3_/CrInSe_3_ heterostructure [29]. However, in our study, we aim to explore the possibility of ferroelectricity through interlayer sliding. This feature was not discussed in the aforementioned reports. We perform a comprehensive first‐principles investigation of the RuO_2_Zn_2_F_2_ bilayer, focusing on the interplay and controllability of sliding ferroelectricity with both A‐type AFM and FM orderings. Since these two magnetic states are nearly degenerate in this system, a feature often overlooked in previous studies, we explicitly consider both phases on equal footing. By systematically analyzing the electronic properties, magnetic interactions, electro‐valley polarization, anomalous Hall response, and piezoelectric properties, our results establish the RuO_2_Zn_2_F_2_ bilayer as a promising platform for next‐generation multiferroic device applications.

## 2 Numerical Method

Theoretical simulations were conducted using the Vienna ab initio simulation package (VASP) [30] with the Perdew–Burke–Ernzerhof (PBE) exchange–correlation functional [31]. To account for the strong electron correlations of Ruthenium (Ru), an effective Hubbard correction of 2.5 eV was applied to the Ru‐4d states [27]. A plane‐wave cutoff energy of 600 eV was employed to determine the self‐consistent charge density, with convergence criteria of 10^−^^6^ eV for total energy and 0.001 eV/Å for atomic forces. The Brillouin zone was sampled using a Monkhorst–Pack *k*‐point mesh of 17 × 17 × 1 for structural relaxation at the PBE level. To incorporate long‐range van der Waals interactions, the DFT‐D2 method of Grimme [32] was used. The dynamic stability of the system was assessed by phonon dispersion calculations using the Phonopy code [33], with a 3 × 3 × 1 supercell. The thermal stability was further evaluated via ab initio molecular dynamics (AIMD) simulations in a 2 × 2 × 1 supercell using the canonical (NVT) ensemble with a Nosé–Hoover thermostat [34]. The electronic band structure was calculated using PBE+U, including the spin–orbit coupling (SOC). The spontaneous electric polarization of RuO_2_Zn_2_F_2_ bilayer was obtained using the Berry phase approach [35]. Finally, the transition energy barrier was determined using the Climbing Image Nudged Elastic Band (CI‐NEB) method [36]. The elastic constants and piezoelectric coefficients were determined using density functional perturbation theory (DFPT) [37]. The intrinsic Berry curvature contribution to the anomalous Hall conductivity (AHC) was obtained using the Wannier interpolation technique. The Wannier functions were constructed from the band structures via the maximally localized Wannier function method [38]. To evaluate the AHC, we employed a numerical tight‐binding model Hamiltonian within the linear‐response Kubo formulism [39]. A denser k‐mesh of 150 × 150 × 1 was used to ensure the accurate real‐space Wannier function construction and reliable AHC calculations.

## 3 Results and Discussion

### 3.1 Symmetry Analysis and Stability

**FIGURE 1:**
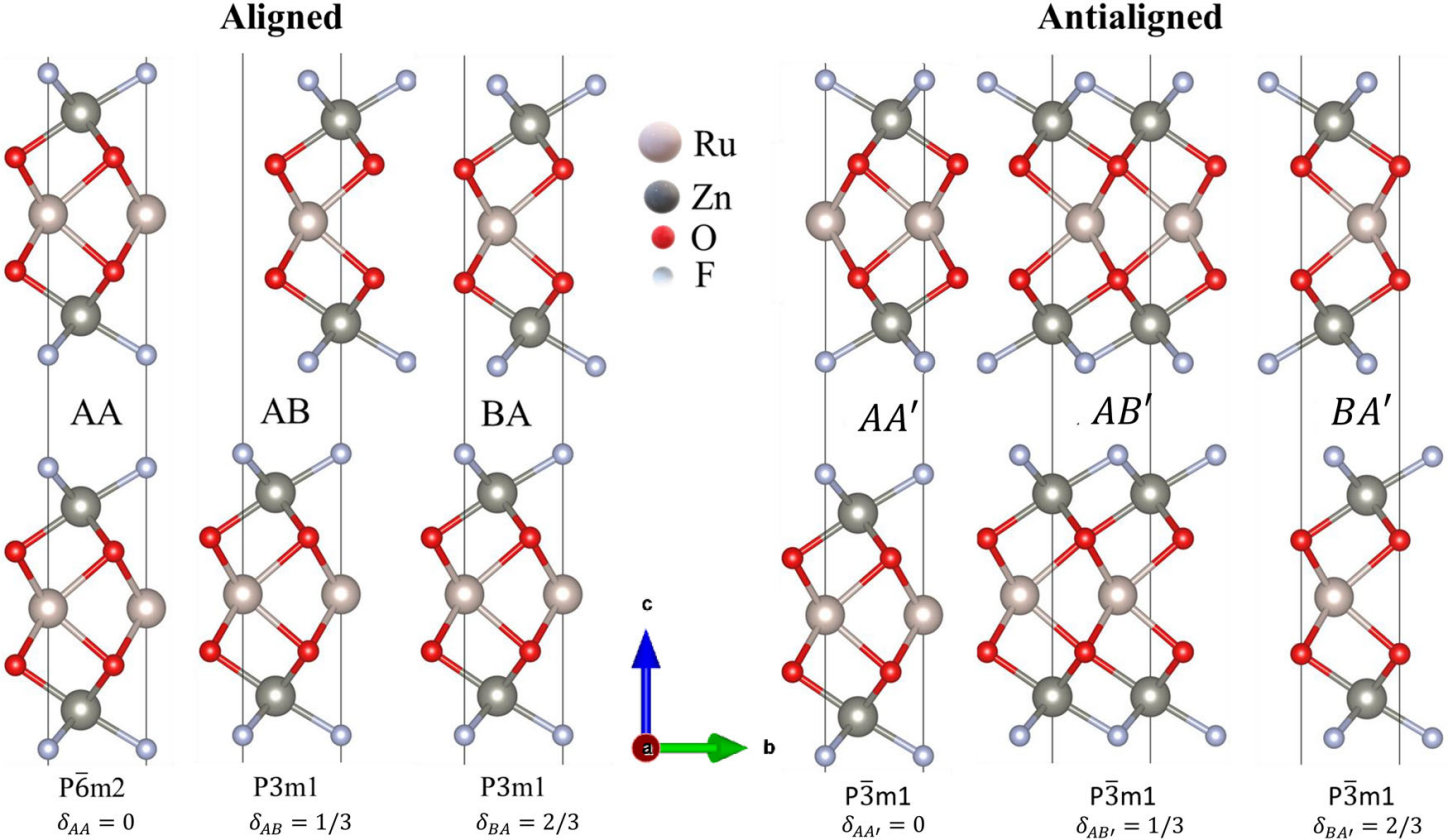
Six possible schematic illustrations of RuO_2_Zn_2_F_2_ bilayers and the corresponding space group.

Figure 1 depicts the side view of aligned and anti‐aligned stacking configurations for the RuO_2_Zn_2_F_2_ bilayer. The bilayer system has six stacking configurations (aligned: AA, AB, BA) and (anti‐aligned: AA′, AB′, BA′). A relative in‐plane displacement (δ) occurs between adjacent layers along the basal (ab) plane; Δ = δ ($\hat{a\;}+\hat{b}$), where $\hat{a\;}$ and $\hat{b}$ are the lattice vectors. Here, $\delta_{AA/AA^{\prime }}=0,\delta_{AB/AB^{\prime }}=\frac{1}{3}\;,$ and $\delta_{BA/BA^{\prime }}=\frac{2}{3}\;$. The aligned bilayer stacking is energetically favorable in the degenerate AB and BA configurations along the polar space group (P3m1, No.156). In contrast, the AA configuration was energetically unfavorable (46 meV/unit cell (u.c) relative to AB stacking) and preserved the space group ($P\bar{6}$ m2, No.187) of the monolayer. Moreover, we found that the anti‐aligned stacking configurations (AA′,  AB′,  BA′) were energetically unfavorable with energy differences of 44, 27, and 25 meV/u.c, respectively. Since the anti‐aligned stackings are both energetically unfavorable, we focus on the aligned stacking in our work. The optimized lattice parameters of the RuO_2_Zn_2_F_2_ bilayer are a = b = 3.10 Å, *α* = *β* = 90°, and *γ* = 120°. The interlayer distance (h) is strongly dependent on the stacking sequence. For instance, the interlayer distance is 3.0 Å in the AA stacking, while it becomes about 2.5 Å in the AB and BA stackings. This trend arises because larger interlayer separations correspond to weaker interlayer binding and vice versa [21]. We also calculated the binding energy (E_b_) of the RuO_2_Zn_2_F_2_ bilayer using the formula *E_b_* = $E_{tot}^{Bilayer}\;-2E_{tot}^{Monolayer}$, where *E_tot_* denotes the total energy. The AB and BA stackings exhibit a binding energy of −1.32 eV/u.c. The negative sign confirms the energetic stability, while the degeneracy reflects the bistability of the two stacking configurations. Besides, we assessed the dynamical stability of the RuO_2_Zn_2_F_2_ bilayer through phonon band structure calculations. The calculated results are shown in Figure S1a. The absence of imaginary frequencies confirms its dynamical stability. We also investigated the mechanical stability of the RuO_2_Zn_2_F_2_ bilayer by calculating its elastic constants (*C_ij_*), obtaining *C*_11_ = 330.41 Nm^−1^, *C*_12_ = 138.12 Nm^−1^, and *C*_66_ = 96.14 Nm^−1^. It is evident that all the elastic constants satisfy the necessary mechanical equilibrium conditions, confirming the mechanical stability of the system [40]. Furthermore, the in‐plane Young's modulus *Y(θ)* and Poisson's ratio *ν(θ)* of the RuO_2_Zn_2_F_2_ bilayer were calculated using the standard formulas for 2D hexagonal systems [41].

(1) $$Y\left(\theta \right)=\frac{C_{11}C_{12}-C_{12}^{2}}{C_{11}\sin^{4}\theta +\left(\frac{C_{11}C_{12}-C_{12}^{2}}{C_{66}}-2C_{12}\right)\sin^{2}\theta \cos^{2}\theta +C_{22}\cos^{4}\theta }$$

(2) $$v\left(\theta \right)=\frac{C_{12}\sin^{4}\theta -\left(C_{11}+C_{12}-\frac{C_{11}C_{12}-C_{12}^{2}}{C_{66}}\right)\sin^{2}\theta \cos^{2}\theta +C_{12}\cos^{4}\theta }{C_{11}\sin^{4}\theta +\left(\frac{C_{11}C_{12}-C_{12}^{2}}{C_{66}}-2C_{12}\right)\sin^{2}\theta \cos^{2}\theta +C_{22}\cos^{4}\theta }$$

where 0° ≤ θ ≤ π/2. We present the orientation‐dependent in‐plane *Y*(*θ*) and *ν*(*θ*) in Figure S1b. The RuO_2_Zn_2_F_2_ bilayer exhibits an in‐plane isotropic Young's modulus of 272.67 Nm^−1^, and this is lower than that found in the graphene (∼340 Nm^−1^) [42]. The high Young's modulus confirms exceptional stiffness. Meantime, Poisson's ratio of 0.41 is also higher than that of the graphene (∼0.17) [42]. This implies that the RuO_2_Zn_2_F_2_ bilayer has ductile behavior with substantial lateral contraction under uniaxial strain. These features suggest robust mechanical stability and strain tolerance, positioning RuO_2_Zn_2_F_2_ bilayer as promising for flexible nanoelectronics, sensors, and resilient 2D devices [43, 44, 45]. Additionally, the thermal stability was further evaluated via ab initio molecular dynamics (AIMD). We present the calculated result in Figure S2a–d. The free energy at 300 K exhibits negligible thermal fluctuations and no structural drift over 4 ps, while the atomic configuration remains intact. This feature may imply thermal stability. In light of the overall structural stability, it is natural to consider the feasibility of a synthetic route to a RuO_2_Zn_2_F_2_ bilayer. Recent advances in fabricating complex 2D layered materials such as septuple‐layer MoSi_2_N_4_ [46] using the chemical vapor deposition method suggest that the proposed RuO_2_Zn_2_F_2_ structure is potentially experimentally accessible. For instance, the distorted h‐RuO_2_ monolayers have already been realized by chemical exfoliation from bulk K‐intercalated RuO_2_ [20]. Furthermore, cryo‐electron microscopy has revealed 2D CaCl crystals with abnormal stoichiometries on reduced graphene oxide membranes, demonstrating that unconventional, isoelectronic halide layers can form under suitable conditions and thereby supporting the plausibility of 2D ZnF‐based layers [47]. Building on these precedents, a practical route to RuO_2_Zn_2_F_2_ begins with an exfoliated h‐RuO_2_ monolayer, which can be symmetrically passivated on both exposed surfaces by ZnF layers through controlled chemical functionalization. Once such ZnF‐terminated RuO_2_ monolayers are obtained, they can be assembled into a RuO_2_Zn_2_F_2_ bilayer via van der Waals stacking or transfer techniques while preserving ZnF passivation at the interface. Drawing on our calculated structural stability together with this established synthesis and stacking methods, we therefore anticipate that RuO_2_Zn_2_F_2_ bilayers can be experimentally realized.

### 3.2 Magnetic Ground State Examination

We considered four possible magnetic configurations of the RuO_2_Zn_2_F_2_ bilayer: ferromagnetic (FM) and three antiferromagnetic (AFM) states in a $1\times \sqrt{3}\times 1$ supercell. Figure S3a–d shows the schematic illustration of the magnetic configurations. We found that the AFM1 is the most stable state among the three AFM configurations. Both AFM2 and AFM3 configurations were highly unstable with a relative energy difference of 713 meV/u.c compared with AFM1. The AFM1 corresponds to the intralayer FM and interlayer AFM arrangement, namely the A‐type AFM configuration. This preference is consistent with the electron‐counting rule for indirect exchange interactions in van der Waals bilayers [48]. However, the energy difference (ΔE) between the FM and AFM1 became merely 0.06 meV/u.c. with a local magnetic moment of 3.36 µ_B_ for the Ruthenium atom. This small ΔE arises from the large interlayer distance ∼10 Å between magnetic sites (See Figure S3e). As a result, the interlayer magnetic coupling can be switched by external magnetic or electric fields [49, 50, 51], magnetic proximity effects [52], or other perturbations [53].

Next, we computed the magnetic anisotropy energy (*MAE*) with spin–orbit coupling (SOC) for the FM and AFM1 states in the AB‐stacked RuO_2_Zn_2_F_2_ bilayer. For both configurations, the out‐of‐plane (z) direction is the easy axis, yielding *MAE*  = *E*_∥_ − *E*_⊥_ ≈ 1.35 meV/u.c. The *MAE* values are nearly equal for FM and AFM1, while AFM1 remains the ground state by an exchange energy of 0.06 meV/u.c, lower than FM. Thus, the uniaxial magnetic anisotropy exceeds the ΔE exchange energy by over an order of magnitude, confirming competing magnetic phases and enabling valley polarization, which requires an out‐of‐plane magnetic easy axis [54]. Since the FM and AFM1 states are energetically degenerated, we consider both magnetic states in the following discussion.

### 3.3 The Interplay Between Sliding Ferroelectricity and Electronic Properties

**FIGURE 2:**
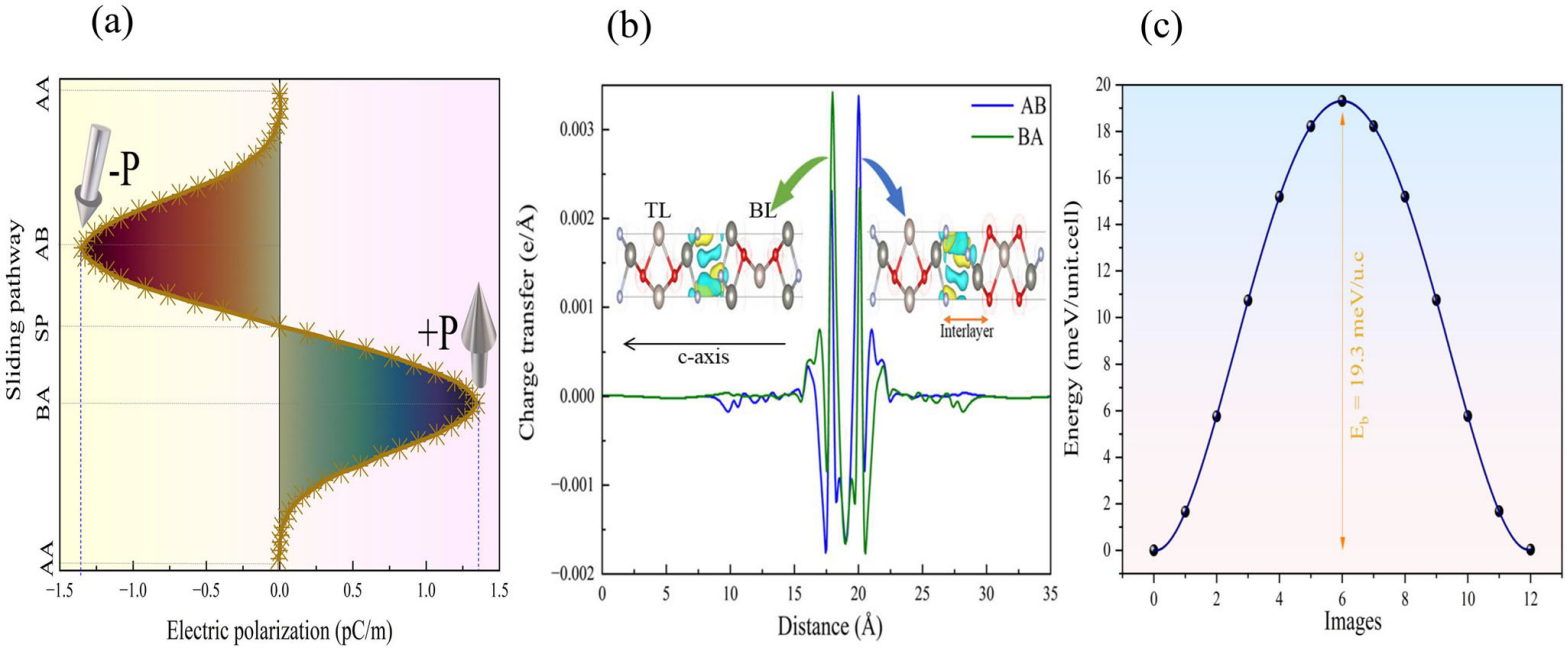
(a) Variation of out‐of‐plane electric polarization along the transition path, (b) charge density differences and plane‐averaged charge density differences along the *c*‐axis in AB and BA stacking of RuO_2_Zn_2_F_2_ bilayer, respectively. The yellow and cyan isosurfaces in the inset represent electron accumulation and depletion, respectively, and (c) the energy barrier between two ferroelectric states.

Sliding ferroelectricity represents a novel manifestation of ferroelectric order in van der Waals multilayers. Here, the electric polarization can be switched not through traditional ionic displacements, but via the relative in‐plane sliding of adjacent atomic layers. In the aligned AA stacking with space group ($P\bar{6}$ m2, No.187) the bilayer hosts three vertical mirror planes related by *C_3_* ​ rotations around the $\hat{a\;}+\hat{b}$ diagonal of the unit cell with $\hat{a}=\left[\begin{array}{c} 1\\ 0 \end{array}\right]\;\;\mathrm{and}\;\;\hat{b}=\left[\begin{array}{c} 1/2\\ \sqrt{3}/2 \end{array}\right]\;$. An additional horizontal mirror plane lies halfway between the two layers. Hence, the stacking lacks polarity and inversion symmetry due to the non‐orthogonality of mirror planes. Note that AB or BA stacking can be achieved by sliding the top layer by $\delta =\frac{1}{3}$, and $\delta =\frac{2}{3}$, along the $\hat{a}$ *+* $\hat{b}$ diagonal direction, respectively. Both stackings belong to the polar space group (*P*3*m*1, No.156). Herein, the three vertical mirror symmetries are preserved with broken horizontal mirror symmetry. This results in a finite out‐of‐plane electric polarization. At the midway (the sliding saddle point, SP, at $\delta =\frac{1}{2}$) between AB and BA stackings, the structure adopts the (Abm2, No.39) space group. This structure retains only a single mirror symmetry through the $\;\hat{a\;}+\hat{b}$ diagonal direction. Moreover, the system becomes invariant under a mirror operation across the plane between layers, combined with a nonsymmorphic translation of $\frac{\hat{a}}{2}+\frac{\hat{b}}{2}$. Thus, any out‐of‐plane electric polarization is forbidden. As a result, only an in‐plane electric polarization along the sliding direction is allowed. For any other translation along the $\hat{a}$ *+* $\hat{b}$ axis, the symmetry is reduced to monoclinic (*Cm*, No.8) with a single mirror plane. This limits the polarization to lie within that plane, but permits both in‐plane and out‐of‐plane components. For sliding paths not aligned with high‐symmetry directions, the system falls into the fully asymmetric (*P*1, No.1) space group. In this case, the polarization may point in any direction. Using the horizontal mirror symmetry of the AA configuration, one finds that the out‐of‐plane electric polarization (OOP) must be an odd function of in‐plane translations. Further enforcing that OOP transforms as a scalar under *C_3_* rotations around the *c*‐axis, we obtain the symmetry‐allowed form,

(3) $$\begin{array}{ccc} P_{\mathrm{OOP}}\;\left(a,b\right) & = & 2P_{\mathrm{OOP}}^{\mathrm{odd}}\sin\left[\pi \left(a+b\right)\right]\left[\cos\left[\pi \left(a-b\right)\right]\right.\\ & & -\left.\cos\left[\pi \left(a+b\right)\right]\right]\; \end{array}$$

where (a,b) are fractional coordinates in sliding‐configuration space defined with respect to the lattice vectors $\hat{a}\;$ and $\hat{b}$, and the mapping between fractional and Cartesian coordinates is given by ℛ = γ(a,b) with $\gamma =(\begin{array}{cc} 1 & 1/2\\ 0 & \sqrt{3}/2 \end{array})\;$. Figure 2a illustrates the interlayer sliding mechanism through the symmetry‐derived polarization landscape, which yields the characteristic butterfly‐type reversal of the out‐of‐plane polarization. For clarity, we emphasize that the axes for sliding displacement and polarization are interchanged in Figure 2a relative to the convention adopted in the symmetry analysis above. However, the underlying symmetry constraints and functional dependence remain unchanged. Thus, the breaking or reinstating inversion symmetry can allow the interlayer charge transfer [15]. Herein, we calculated the charge density differences as Δρ = ρ_bilayer_−ρ_top_−ρ_bottom_. Figure 2b shows the plane‐averaged charge density difference along the *c*‐axis, and we found the opposite charge‐transfer behavior for the two stacking configurations. In **AB stacking**, the electron accumulation (yellow isosurface) occurs in the top layer (TL), while the electron depletion (cyan isosurface) is observed in the bottom layer (BL). This implies a net charge transfer from the BL to TL, and this results in an electric polarization along the −*c* axis and vice versa in the case of BA stacking. The broken mirror symmetry and unequal interfacial charge redistribution induce a vertical electric polarization of 1.35 pC/m in the RuO_2_Zn_2_F_2_ bilayer, which can be modulated by stacking variations. The magnitude of the electric polarization of the RuO_2_Zn_2_F_2_ bilayer is comparable to that of the Janus MoSiGeN_4_ bilayer (1.40 pC/m) [55] and exceeds that of RuX_2_ (X = Cl/Br, 0.20/0.21 pC/m) [56] and VX_2_P_4_ (X = Si/Ge, 0.42/0.87 pC/m) [56]. Note that the magnitude of the electric polarization is equal in both FM and A‐type AFM states. This implies that the ferroelectric behavior is determined by the stacking configuration rather than the magnetic order. Remarkably, the energy barrier between the two polar configurations (AB and BA) is merely 19.3 meV/u.c (Figure 2c). This indicates that the reversal of electric polarization can be efficiently realized via interlayer sliding. Such a low barrier suggests minimal energy consumption during polarization switching, enabling ultrafast operation and reduced power usage, key attributes for next‐generation ferroelectric devices and nonvolatile memory technologies.

**FIGURE 3:**
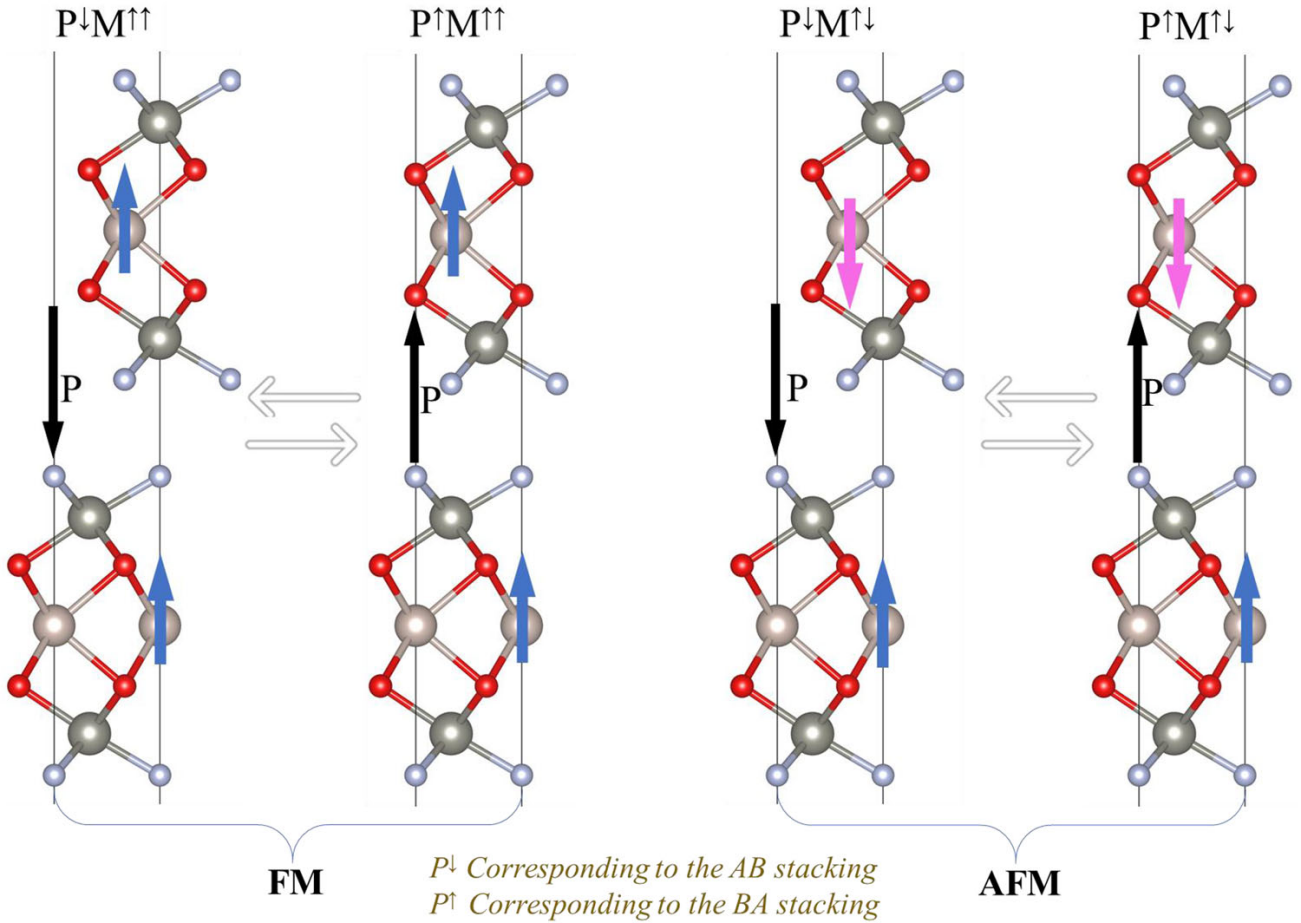
Four different Ferroelectric/Magnetic configurations of bilayer RuO_2_Zn_2_F_2_. The black, blue, and pink arrows represent FE polarization, spin up, and spin down, respectively.

Now, we investigate the interplay between valley polarization and magnetoelectric coupling in the RuO_2_Zn_2_F_2_ bilayer. We construct two ferroelectric/ferromagnetic (FE/FM) states with positive and negative electric polarization (P^↑^M^↑↑,^ P^↓^M^↑↑^) and similarly two ferroelectric/antiferromagnetic (FE/AFM) states (P^↑^M^↑↓^, P^↓^M^↑↓^). Here, *P* and *M* denote the electric polarization and magnetization. We present the schematic illustration in Figure 3. The blue, pink, and black colors represent the spin‐up, spin‐down components, and electric polarization direction, respectively.

**FIGURE 4:**
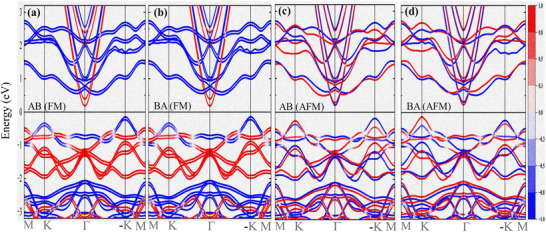
(a‐d) Spin‐projected band structure of bilayer RuO_2_Zn_2_F_2_ with SOC. Red and blue colors represent spin up and spin down weights, respectively.

Figure 4a–d displays the spin‐projected band structures of the RuO_2_Zn_2_F_2_ bilayer, including spin–orbit coupling for (FE/FM) and (FE/AFM) configurations. We observed that the degeneracy of +K and ‐K valleys is lifted near the valence band maximum (VBM) through spin‐momentum locking, leading to a spontaneous valley polarization phenomenon. The valley polarization is defined as $\Delta E_{V}=E_{V}^{+K}\;-E_{V}^{-K}$. For the FE/FM states, we obtained identical band structures for both positive and negative electric polarizations. Both configurations display bipolar semiconductor characteristics with an indirect band gap of 0.33 eV, as shown in Figure 4a,b. This indicates that switching the electric polarization does not affect the ferromagnetic order. Besides, we obtained a large valley polarization of −190 meV, and this is comparable to that reported in the RuO_2_(ZnF)_2_ monolayer in the ferromagnetic state. Notably, the valley polarization in our bilayer system is approximately three times larger than that of previously reported VSi_2_N_4_ and VSi_2_P_4_ monolayers [57, 58]. In contrast, in the FE/AFM states, we obtained a strong interplay. The P^↓^M^↑↓^ configuration (AB stacking) produces a monopolar semiconductor (with a band gap of 0.4 eV) in the spin‐down channel with ΔE_V_ = −122 meV, while the sign of valley polarization is reversed with the same magnitude in P^↑^M^↑↓^ (BA stacking). Furthermore, the spin states at the K and ‐K valleys satisfy the relation, $E_{AB}^{S^{↑}}\left(K\right)=E_{BA}^{S^{↓}}\;\left(-K\right)$. Besides, in CBM, we found spin rearrangement, e.g., the spin‐up becomes the spin‐down at the +K valley with sliding as shown in Figure 4c,d. This reversal of spin results in a complete exchange of the spin‐polarized CBM at the +K and −K valleys. Overall, these results demonstrate that the electric polarization switching induces a 180° reversal of magnetic moments in both layers to maintain the global band structure, establishing ferrovalley coupling as the underlying mechanism linking ferroelectric and magnetic orders.

### 3.4 The Anomalous Hall Conductivity in the RuO_2_Zn_2_F_2_ Bilayer

In multiferroic and ferrovalley systems, the anomalous Hall effect (AHE) serves as a key transport signature of the interplay among spin–orbit coupling, broken inversion symmetry, and magnetic order. Therefore, we also computed the anomalous Hall conductivity (AHC). Within the Berry phase framework and linear response Kubo formalism, the AHC (σ_xy_) can be expressed as:

(4) $$\sigma_{xy}=-\frac{e^{2}}{\hbar }\sum_{n}\int_{BZ}^{BZ}\frac{dk}{{\left(2\pi \right)}^{3}}f_{n}\left(k\right)\Omega_{n,z}\left(k\right)$$

where *e*, *f_n_*, and Ω_*n*,*z*_(*k*) represents the elementary charge, the Fermi‐Dirac distribution function, and Berry curvature. The Berry curvature is given by,

(5) $$\Omega_{n,z}\left(k\right)=-2Im\sum_{m\neq n}\frac{\langle u_{nk}|v_{x}|u_{mk}\rangle \langle u_{mk}|v_{y}|u_{nk}\rangle }{{\left(E_{mk}-E_{nk}\right)}^{2}}$$

**FIGURE 5:**
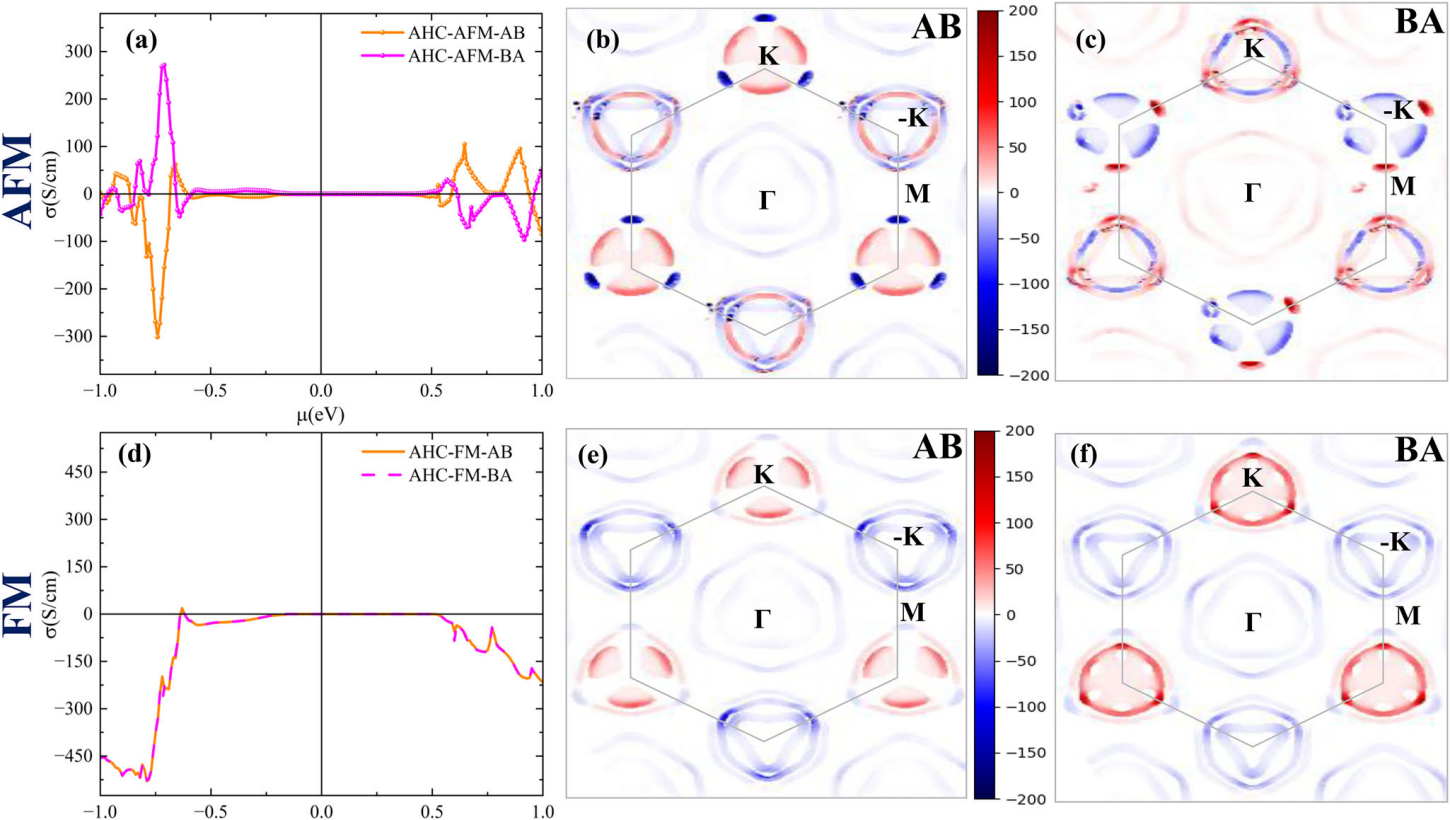
Anomalous Hall conductivity as a function of chemical potential for AB and BA stackings in the (a) AFM and (d) FM spin states. The corresponding Berry curvature distributions over the 2D Brillouin zone are shown for AB and BA stackings in the AFM state ((b) and (c)) and in the FM state ((e) and (f)), respectively.

Here, *v_x_* and *v_y_* are the velocity operators, 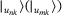 are the periodic parts of the wave functions, and *E_nk_(E_mk_)* are the corresponding eigenvalues. The Berry curvature was computed using the Wannier interpolation technique [59]. First, a set of maximally localized Wannier functions (MLWFs) was generated from the Bloch wave functions. A Fourier‐transformed Wannier Hamiltonian was then constructed by projecting Bloch states onto these MLWFs. This Hamiltonian allows an efficient and accurate evaluation of the Berry curvature across the Brillouin zone. Figure 5a shows the anomalous Hall conductivity (AHC) for the AFM spin configuration in both AB and BA stacking orders. We obtained a large p‐type AHC of −301 S/cm at −0.74 eV for AB stacking, while it became 271 S/cm at −0.71 eV in BA. To elucidate the origin of these contrasting behaviors, we analyzed the Berry curvature distribution for both stacking orders in the AFM spin configuration. Figure 5b,c show the Berry curvature across the 2D Brillouin zone (BZ) for AB and BA stackings at −0.74 and −0.71 eV, respectively. The BZ is outlined by the gray solid lines and labeled with the high‐symmetry points Γ, M, K, and ‐K. Positive and negative Berry curvature contributions are denoted by red and blue regions, which collectively determine the sign and magnitude of the AHC. In the AB stacking, the negative Berry curvature is centered at Γ along with partial negative contributions near the ‐K valleys. This negative contribution outweighs the positive contribution from the K points, resulting in a large negative AHC of −301 S/cm. Conversely, in the BA stacking, the positive Berry curvature contributions at Γ and the K valleys dominate the negative from ‐K points, giving rise to a large positive AHC of 271 S/cm. Furthermore, we also investigated the AHC in the FM spin configuration. Figure 5d–f show the AHC and the corresponding Berry curvature distributions over 2D BZ for both AB and BA stackings in the FM state. Both stackings exhibit a very large p‐type AHC of −527 S/cm at −0.79 eV. In the FM configuration, the K valleys contributed positively. However, the dominant negative contributions originated from the −K valleys and the Γ point. These negative Berry curvature contributions prevail in both AB and BA stackings, ultimately leading to a very large negative AHC. Note that the large AHC is found via hole doping of approximately 1.19 × 10^13^ cm^−2^ in the AB‐stacked bilayer, and this carrier concentration can be experimentally achievable through electrostatic gating [60, 61, 62, 63]. This doping induces metallicity (Figure S4a,b) without compromising structural stability or magnetic order. The out‐of‐plane MAE of 2.03 meV/u.c remains robust in both magnetic states under doping. Note that ferroelectricity is strongly suppressed and often destroyed in metallic systems due to free carriers screening effect of an internal electric field. However, it has been reported that ferroelectricity can be found in van der Waals‐type metal, like in few‐layer WTe_2_ [64]. A detailed investigation of this aspect is therefore deferred to future work. Without loss of generality, we focus here on the pristine RuO_2_Zn_2_F_2_ bilayer. The strong spin–orbit‐coupling‐driven symmetry breaking and ferrovalley interactions position this system as a promising platform for nonvolatile spintronic and valleytronic applications.

### 3.5 Large Piezoelectricity in the RuO_2_Zn_2_F_2_ bilayer

Since the RuO_2_Zn_2_F_2_ bilayer belongs to the polar C_3v_ point group, we extended our study to the piezoelectric effect to quantify the electric polarization generated by small deformations. Using density functional perturbation theory (DFPT), we calculated the stress and strain piezoelectric tensors: *e_ij_* and *d_ij_* as follows [65]:

(6) $$C_{ij}=\left\{\begin{array}{ccc} C_{11} & C_{12} & 0\\ C_{12} & C_{11} & 0\\ 0 & 0 & \frac{C_{11}-C_{12}}{2} \end{array}\right\}$$

(7) $$e_{ij}=\left\{\begin{array}{ccc} e_{11} & -e_{11} & 0\\ 0 & 0 & -\frac{e_{11}}{2}\\ e_{31} & e_{31} & 0 \end{array}\right\}$$

(8) $$d_{ij}=\left\{\begin{array}{ccc} d_{11} & -d_{11} & 0\\ 0 & 0 & -d_{11}\\ d_{31} & d_{31} & 0 \end{array}\right\}$$

(9) $$d_{11}=\frac{e_{11}}{C_{11}-C_{12}}\;\;\mathrm{and}\;\;d_{31}=\frac{e_{31}}{C_{11}+C_{12}}\;$$

We find that the RuO_2_Zn_2_F_2_ bilayer has exceptional piezoelectric properties with stress coefficients of (*e*_11_ = 15.6 × 10^−10^ C/m and *e*_31_ = 2.52 × 10^−10^ C/m) and strain coefficient of (*d*_11_ = 8.13pm/V and *d*_31_ = 0.61 pm/V). Note that the h‐BN has *e*_11_ = 1.4 × 10^−10^ C/m, *d*_11_ = 0.6 pm/V [66], while the MoS_2_ has *e*_11_ = 3.6 × 10^−10^ C/m, *d*_11_ = 3.5 pm/V [67]. Besides, both Cr_2_SeO [17] and V_2_SeTeO [68] altermagnets have *e*_11_ = 1.18 × 10^−10^ C/m and *d*_31_ = 0.245 pm/V, respectively. Compared with these systems, the RuO_2_Zn_2_F_2_ bilayer shows substantially larger values. This pronounced piezoelectric response arises from the large electronegativity difference between the constituent atoms, and this feature induces strong dipole polarization across the interface. These findings may suggest that the RuO_2_Zn_2_F_2_ bilayer can be a promising candidate material for applications in nanoscale energy harvesting, sensing, and electromechanical devices.

## 4 Conclusion

In conclusion, we performed ab initio calculations to predict and understand the interplay between sliding ferroelectricity and magnetism in RuO_2_Zn_2_F_2_ bilayers. Our results reveal that the RuO_2_Zn_2_F_2_ bilayer hosts closely competing ferromagnetic and A‐type antiferromagnetic ground states. The RuO_2_Zn_2_F_2_ bilayer has a band gap of 0.40 eV in the A‐type antiferromagnetic state, while it has a band gap of 0.33 eV in the ferromagnetic state. Both AFM1 and FM states exhibit a large out‐of‐plane magnetic anisotropy of 1.35 meV/u.c., indicating strong spin–orbit‐driven uniaxiality. A spontaneous electric polarization of 1.35 pC/m arises from interlayer charge transfer, accompanied by an ultralow energy barrier of 19.3 meV/u.c., which is advantageous for fast‐switching and non‐volatile memory applications. Moreover, our systematic investigation shows a strong stacking‐ and spin‐configuration‐dependent anomalous Hall conductivity (AHC). In the AFM state, we found that AB and BA stackings exhibit large but opposite AHC values of −301 S/cm and +271 S/cm due to contrasting Berry curvature contributions at the Γ point and K valleys. In the FM state, both stackings display a remarkably large AHC of −527 S/cm at −0.79 eV. Additionally, we obtained outstanding piezoelectric coefficients (*e*_11_ = 15.6 × 10^−^^10^ C/m, *e*_31_ = 2.52 × 10^−^^10^ C/m, *d*_11_ = 8.13 pm/V, and *d*_31_ = 0.61 pm/V), and this is superior to those of many existing 2D materials. These exceptional ferroic, electronic, and piezoelectric properties position RuO_2_Zn_2_F_2_ as a multifunctional material with strong potential for valleytronic, spintronic, energy harvesting, and sensing applications, opening new avenues for next‐generation nanoelectronic devices.

## Author Contributions

D.B., I.K., and G.S. contributed to writing the original draft, data curation, software development, and formal analysis. J.H. was responsible for review and editing, validation, supervision, project administration, funding acquisition, and conceptualization.

## Conflicts of Interest

The authors declare no conflicts of interest.

## Supporting information




**Supporting File**: advs74923‐sup‐0001‐SuppMat.docx.

## Data Availability

The data that support the findings of this study are available from the corresponding author upon reasonable request.
