# Supplementary material for: Interlayer‐Sliding‐Enabled Multiferroicity and Giant Switchable Anomalous Hall Conductivity in RuO2Zn2F2 Bilayer
Source: Adv Sci (Weinh). 2026 May 7:e74923. Online ahead of print. doi: 10.1002/advs.74923 (PMC13325646; doi:10.1002/advs.74923)
Supplement: Supplementary file 1 — Supporting File: advs74923‐sup‐0001‐SuppMat.docx. [file ADVS-9999-e74923-s001.docx]

**Supporting information**

**Interlayer-Sliding-Enabled Multiferroicity and Giant Anomalous Hall Conductivity in RuO_2_Zn_2_F_2_ Bilayer**

*Djamel Bezzerga*^†^*, Imran Khan*^†^*,* *Ganie Suhail Ahmad*^†^*, and Jisang Hong*^*^

Department of Physics, Pukyong National University, Busan 48513, South Korea.


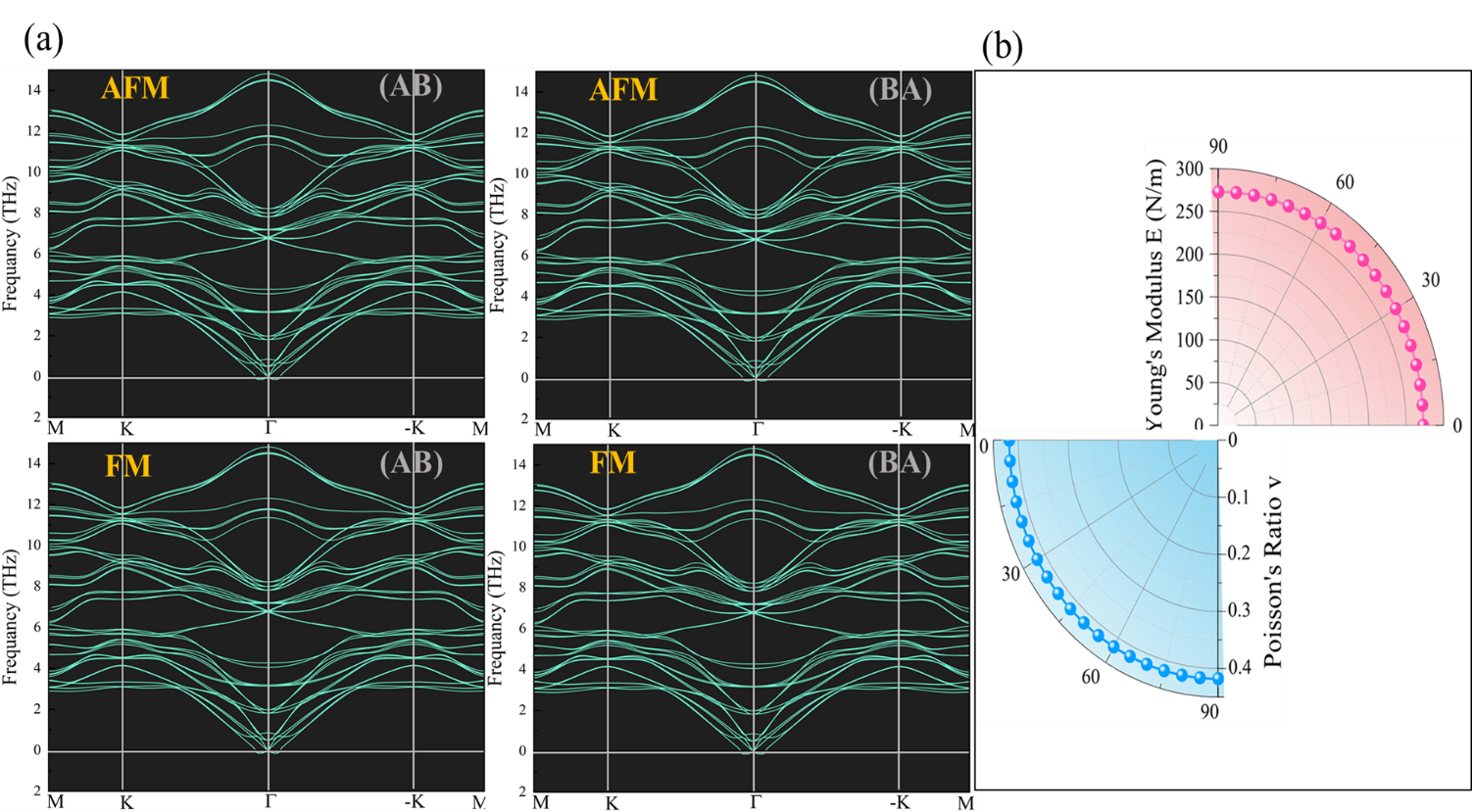


**Figure. S1** (a) Phonon dispersions of the RuO_2_Zn_2_F_2_ bilayers for (AB and BA stackings for AFM and FM orders) (b) The spatial Young modulus and Poisson ratio.


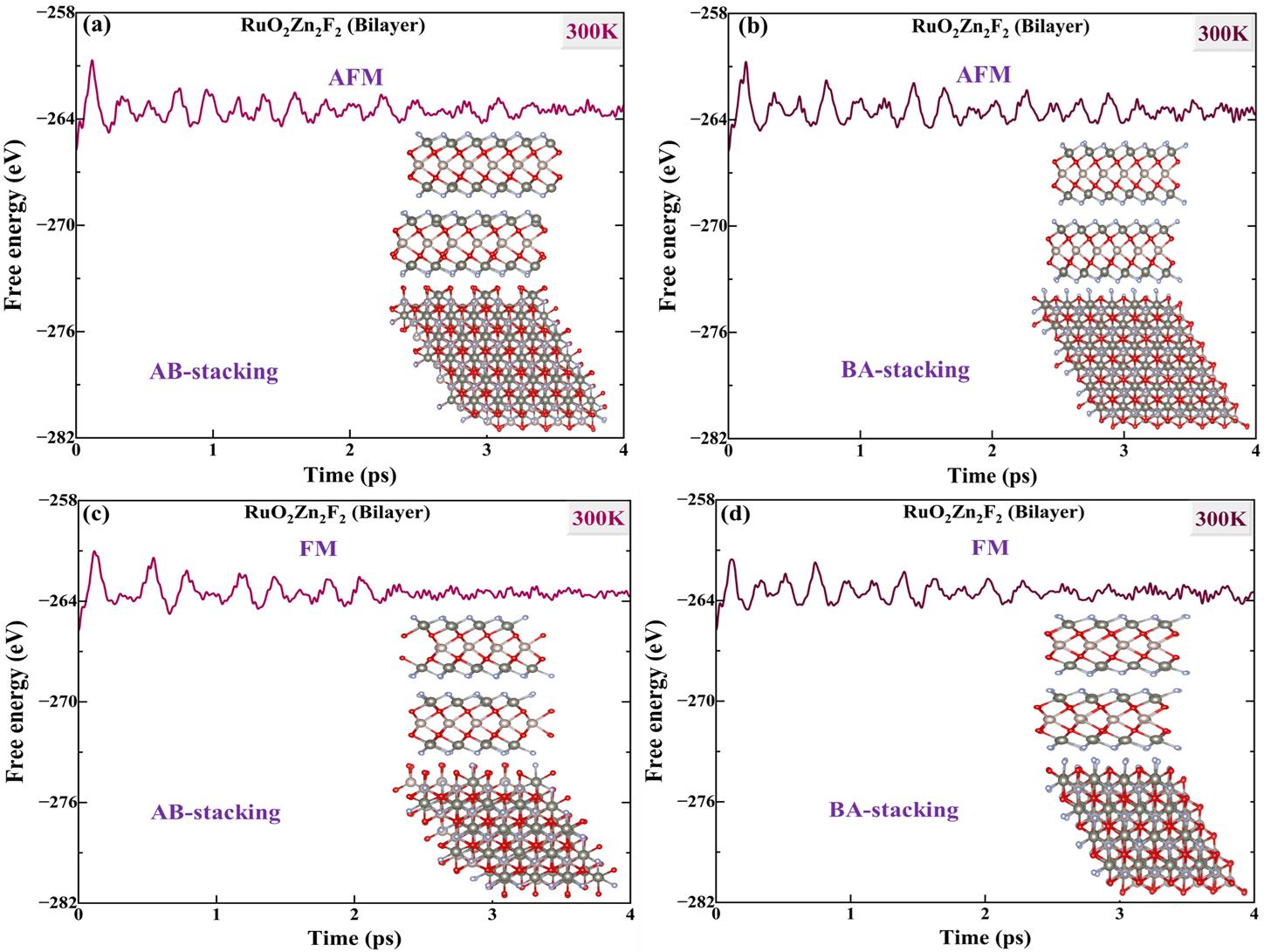


**Figure. S2** (a-d) The total energy fluctuations with respect to the simulation time of RuO_2_Zn_2_F_2_ bilayer (AB and BA stackings for AFM and FM orders) at 300 K as well as the corresponding structure snapshots at the end of 4.0 ps AIMD simulations.

**
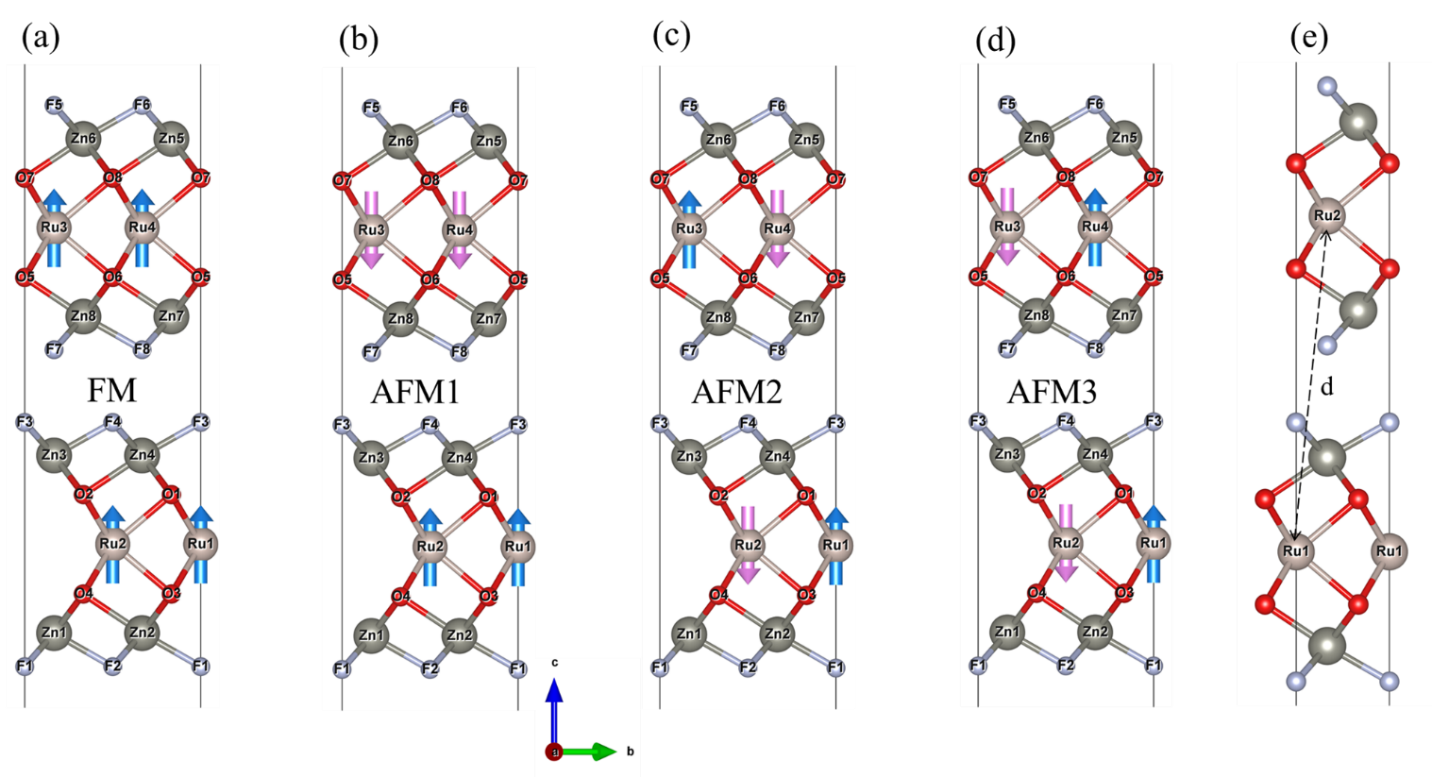
**

**Figure. S3** (a-d) Different magnetic configurations of RuO_2_Zn_2_F_2_ bilayers (e) the interlayer distance between Ru atoms.


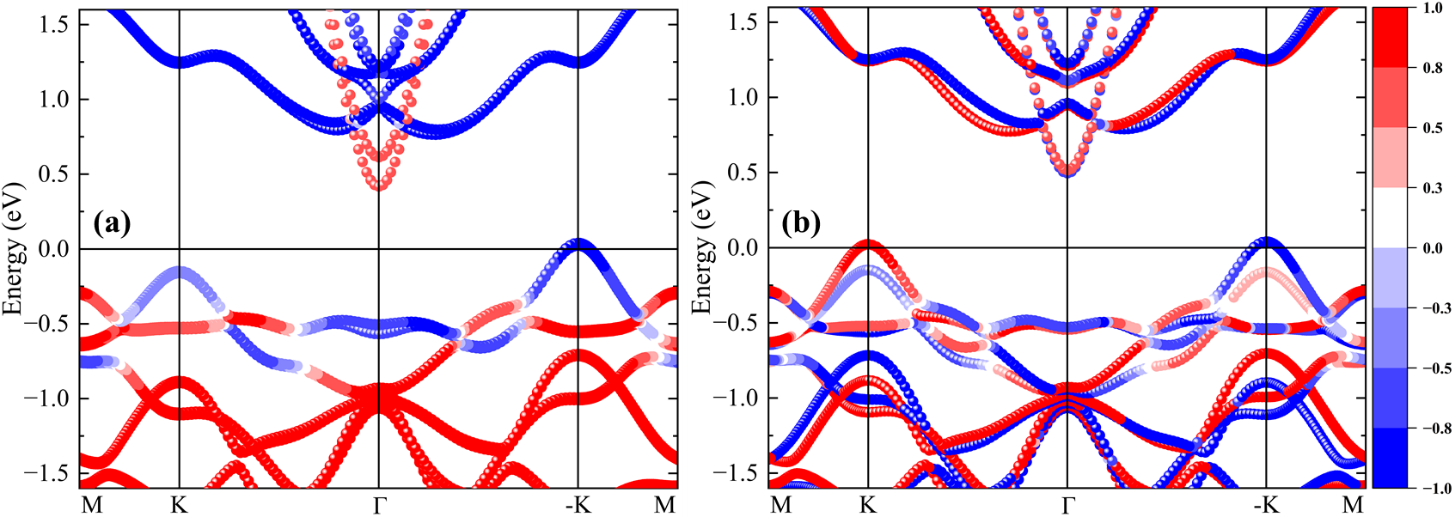


**Figure S4.** Spin projected band structure including spin-orbit coupling for (a) AFM state and (b) FM state in AB stacking for hole doping concentration of 1.19 × 10^13^ cm^-2^.
